# Dysbiosis of gut and urinary microbiota in urolithiasis patients and post-surgical cases

**DOI:** 10.3389/fcimb.2025.1633783

**Published:** 2025-08-13

**Authors:** Yuanyuan Jia, Meng Han, Haoyu Ge, Jie Qiao, Ruyan Chen, Chenyu Li, Cailin Liu, Lei Fang, Yanhao Shen, Saiqi Qi, Yuli Wang, Xiaobing Guo

**Affiliations:** ^1^ Department of Laboratory Medicine, The First Affiliated Hospital of Zhengzhou University, Zhengzhou, China; ^2^ Department of Clinical Laboratory, The First Affiliated Hospital, and College of Clinical Medicine of Henan University of Science and Technology, Luoyang, China; ^3^ Department of Laboratory Medicine, Zhengzhou Central Hospital Affiliated to Zhengzhou University, Zhengzhou, China

**Keywords:** gut microbiota, urinary microbiota, urolithiasis, kidney stones, post-surgical

## Abstract

**Background:**

Research on the microbial roles in urolithiasis primarily focuses on the intestinal microbiota. This study analyzed urine and fecal samples from three cohorts: healthy controls (Control), patients with urinary stones (US), and postoperative patients (PS). We conducted 16S rRNA sequencing analysis to evaluate the variations in microbial communities among these groups during urinary stone production and therapy processes.

**Results:**

In fecal microbiota, alpha diversity was lower in the stone group versus the control group, with the postoperative group showing the lowest diversity. The β diversity analysis revealed some differences in the microbial community structure of individuals with different health conditions. LEfSe and Wilcoxon analyses were utilized to discover species that exhibited significant differences between groups. *Enterobacteriaceae* and *Bacteroides* are more abundant in patients with stones. The increased abundance of *Lactobacillus*, *Lachnospiraceae*, *Rumenococcaceae*, *Faecalibacterium*, and *Prevotella* is associated with a reduced risk of kidney stones.

**Conclusions:**

Alterations in urinary and intestinal microbiota may indicate urolithiasis status and treatment response. Future studies should explore microbiota modulation (e.g., probiotics) as an adjunctive strategy, while antibiotic stewardship is warranted to minimize microbiota disruption.

## 1 Introduction

Urinary tract stones (UTS), also known as urolithiasis, are a common disease of the urinary system. Upper urinary tract stones (UUTS) mainly consist of nephroliths and ureteroliths. Approximately 80% of kidney stones are calcium stones, which encompass calcium oxalate and calcium phosphate stones, while the remaining categories include struvite stones, uric acid stones, and cystine stones (Peerapen and Thongboonkerd, 2023; Jebir and Mustafa, 2024). Ureteral stones are generally produced by the passage of kidney stones into the ureters, so the incidence of ureteral stones is less than that of renal stones (Li et al., 2019; Tan et al., 2024). Research suggests that the incidence of kidney stones is 7.80% (95% CI 5.8-10.0) in China and 11.0% (95% CI 10.1-12.0) in the United States, with men exhibiting a higher risk of developing stones compared to women (Wang et al., 2017; Ferraro et al., 2022b; Hill et al., 2022; Tan et al., 2024). Various factors contribute to the formation of kidney stones, including gender, age, dietary habits, genetics, environmental influences, lifestyle (e.g., obesity), climate change, and comorbidities (e.g., hypertension, diabetes) (Shin et al., 2018; Maddahi et al., 2020; Ferraro et al., 2022a). The prevalence of nephrolithiasis has been progressively rising throughout the years; notably, there exists a five-year recurrence risk estimated at 40% (Worcester and Coe, 2010; New and Somani, 2016). Urolithiasis significantly affects individuals and society, putting financial burdens on patients and affecting their quality of life (Geraghty et al., 2020; Ní Néill et al., 2023).

In recent years, the correlation between microbiome and human health has garnered heightened attention. The microbial community colonized in the human intestine is called intestinal microbiota. Obesity, gastrointestinal diseases, cardiovascular diseases, and some neurological diseases are linked to disturbances in the composition and function of the gut microbiota (Cook and Mansuy-Aubert, 2022; Hu et al., 2022; Maffei et al., 2022; Ahmadi et al., 2024). Recent studies have demonstrated that *Oxalobacter formigenes*, a Gram-negative bacterium found in the gut, degrades oxalate, consequently decreasing urine oxalate excretion and mitigating the risk of kidney stone formation (Mehta et al., 2016; Yuan et al., 2023). Furthermore, the administration of antibiotics and probiotics (such as *Lactobacillus* and *Bifidobacterium*) can modulate the gut microbiota, thereby affecting the formation of kidney stones (Tasian et al., 2018; Joshi and Goldfarb, 2019; Liu et al., 2021b). The imbalance of the urinary microbiota is likewise linked to urolithiasis (Razi et al., 2024). Urease-producing bacteria are associated with the development of infectious stones, while *Escherichia coli*, a non-urease-producing bacterium, is the most common bacterium found in the urine samples of individuals with stone formation (Amimanan et al., 2017; Dutov et al., 2018; Ranjit and Singh, 2020). Recently, Anna Zampini et al. used multi-point, multi-omics research methods to demonstrate that the urinary tract microbiome exhibits a stronger connection with urinary stone illness than the gut microbiota (Zampini et al., 2019). Therefore, we need to examine the function of bacteria in stone production and treatment by evaluating both gut and urine microbiota.

Microorganisms play a crucial role in the formation of stones. Kait F. Al et al. demonstrated that changes in multiple microbiota may indicate the production of kidney stones (Al et al., 2023). What’s more, on account of the widespread use of antibiotics, multidrug-resistant bacteria have emerged and become widespread, which poses significant challenges for the clinical treatment of stone disease. The analysis of urethral and intestinal flora associated with urinary tract stones can serve as a valuable marker for evaluating disease development. Prior research has demonstrated that the microbiota of individuals with stones is significantly different from that of healthy individuals. However, there is limited research on the changes in the microbiota before and after kidney stone surgery. In this study, we collected fecal and urine samples from the healthy, stone, and postoperative groups for 16S rRNA sequencing analysis. The results were analyzed to assess the influence of intestinal and urinary microbiota on stone disease.

## 2 Materials and methods

### 2.1 Participants

Patients were mainly recruited from those who visited the urology department of a tertiary hospital in Zhengzhou, Henan province, from September 2021 to May 2022. The criteria for inclusion are as follows: (1) Age 10 to 70 years old; (2) Imaging diagnosis of kidney stones or ureteral stones. The criteria for exclusion are as follows: (1) Report the use of antibiotics within 2 weeks of enrollment; (2) The patient has a history of gastrointestinal disease or metabolic disease; (3) Infection from other sites or sample contamination; (4) Failure of the nucleic acid quality test. The effective specimens obtained were grouped. We collected urine and stool samples from healthy people, stone patients, and patients after stone surgery, and named the urine samples as group 1 and the stool samples as group 2. The urine and fecal specimens of patients diagnosed with UUTS were US1 and US2 (n1 = 17, n2 = 29), the urine and fecal specimens from patients after stone surgery respectively, were PS1 and PS2 (n1 = 6, n2 = 10), and urine and fecal samples from healthy individuals were Control1 and Control2 (n1 = 22, n2 = 26). In addition, urine was collected for urine chemical analysis, with Control (n=31) in the healthy group, US (n=38) in the stone group, and PS (n=12) in the postoperative stone group.

### 2.2 Sample collection

Patients should abstain from eating or drinking for 6 hours before specimen collection. Collect fresh feces of subjects about 3–5 g into special containers. Before collecting clean midstream urine from the test subject, the urethral opening and surrounding skin should be cleaned to avoid contamination with vaginal discharge, semen, etc. Subsequently, the initial portion of urine should be discarded, and the midstream urine should be collected in a non-contaminated urine container. Dispense the sample into 1.5 ml low adsorption tubes and mark the sample name. Seal the sample tubes with parafilm membrane and store them at -80°C before sending them for inspection.

### 2.3 Sample processing

Use a DNA extraction kit to extract DNA from samples. The HiPure Stool DNA Kit and HiPure Bacterial DNA Kit were used to extract stool samples and urine samples, respectively. DNA concentration was assessed using the M9. Equalbit 1xdsDNA HS Assay Kit. 20–50 ng of DNA was utilized to produce amplicons encompassing the V3 and V4 hypervariable regions of the bacterial 16S rRNA gene. The forward primer contains the sequence ‘ACTCCTACGGGAGGCAGCAG’ and the reverse primer contains the sequence ‘GGACTACHVGGGTWTCTAAT’. In addition, an index-bearing adapter is added to the end of the PCR product of 16S rDNA by PCR for NGS sequencing. The concentration is measured using a microplate reader (Tecan, Infinite 200 Pro), while the fragment size is analyzed by 1.5% agarose gel electrophoresis, anticipated to be approximately 600 bp. Ultimately, PE250/FE300 paired-end sequencing was conducted by the Illumina MiSeq/Novaseq (Illumina, San Diego, CA, USA) equipment instructions to acquire Pass Filter Data.

### 2.4 Quality control

The samples of this experiment were processed in a sterile experimental environment and under controlled conditions, stored at -80°C, and sent for testing. All samples were sequenced under the same experimental conditions and instruments.

### 2.5 Sequencing data processing

The forward and reverse reads acquired during paired-end sequencing are initially spliced in pairs, filtering out the sequences containing N and retaining the sequences longer than 200 bp. After quality filtering and removal of the chimera sequence, the final sequences were used for OTU clustering. Sequence clustering was performed using VSEARCH (1.9.6) (sequence similarity set to 97%), and the 16S rRNA reference database (Silva 138) was used for alignment. Then, the RDP classifier (Ribosomal Database Program) Bayesian algorithm was used to analyze the species taxonomy of the representative sequences of OTU, and the community composition of each sample was counted under different taxonomic levels.

### 2.6 Bioinformatics analysis and statistical tests

The data was subjected to statistical analysis with SPSS. When the assumptions of normality and homogeneity of variance were met, either a T-test or ANOVA was employed. For the two sets of data, if they follow a normal distribution but exhibit unequal variances, the corrected t-test is employed; when neither normality nor homogeneity of variance is satisfied, the Wilcoxon rank-sum test is utilized. For three or more sets of data, when normality or homogeneity of variance is not met, the Kruskal-Wallis rank-sum test is employed.

In this study, the Kruskal-Wallis test with Bonferroni-adjusted *post hoc* tests was applied to urinary electrolyte data. We compared alpha diversity indices in the healthy, stone, and postoperative groups, including Chao1, ACE, Shannon, and Simpson, to assess species richness and distribution uniformity in the samples. The coverage index was used to determine whether the sequencing depth covers the entire bacterial diversity. Alpha diversity between groups was compared using the Kruskal-Wallis rank sum and the Wilcoxon rank-sum test. We assessed beta diversity among three groups by principal coordinate analysis (PCoA), NDMS, and Adonis test. LEfSe and Wilcoxon rank-sum tests were employed to identify differentially abundant species, with FDR adjustment applied to Wilcoxon comparisons, using a linear discriminant analysis (LDA) score threshold of 3.0 for LEfSe.

## 3 Result

We successfully collected US1 and US2 (n1 = 17, n2 = 29), PS1 and PS2 (n1 = 6, n2 = 10), and Control 1 and Control 2 (n1 = 22, n2 = 26) for sequencing analysis. A total of 8183577 16S rDNA fragments were obtained from intestinal and urinary tract flora by 16S rRNA gene sequencing. After removing chimeric sequences, 6930657 valid sequences were obtained for further analysis, with an average read length of about 452.6 bp (**Supplementary Figure 1**).

### 3.1 Urine electrolyte analysis

We collected urine samples from the subjects for urine chemical analysis and calculated the median and quartile for each group. The nonparametric Kruskal-Wallis H test showed that *P <*0.05 for Calcium (Ca) and Phosphorus (P), which was statistically significant, indicating that there was a statistically significant difference between Ca and P among the three groups (**Table 1**). The results showed that the Ca elements of the healthy group and the stone group were both *P <*0.05 compared with the postoperative group, respectively, and there was a statistical difference. The P element of the healthy group was significantly different from that of the postoperative group (*P <*0.05).

**Table 1:** Statistical analysis of urine chemical elements in healthy, stone, and postoperative groups.

Element	Control (N=31)	US (N=38)	PS (N=12)	H	*P*
K	36.20 (8.49,60.04)	24.09 (12.68,28.95)	19.15 (10.02,39.89)	2.388	0.303
Na	139.00 (38.00,196.00)	107.00 (67.50,137.75)	90.00 (78.75,133.75)	1.793	0.408
Cl	109.50 (33.00,183.40)	81.25 (57.30,125.08)	83.30 (50.73,107.35)	2.249	0.325
Ca	2.36 (0.92,3.34)^a^	1.71 (1.12,3.88)^b^	4.33 (2.74,5.49)	8.141	0.017
P	13.37 (4.26,14.73)^c^	13.35 (9.88,15.29)	18.69 (13.50,25.48)	10.214	0.006
Mg	1.77 (0.75,3.03)	2.27 (1.25,2.91)	2.61 (2.07,3.30)	3.363	0.186

The median and quartile of each group, the test statistic H, and *P* values of the Kruskal-Wallis test were calculated separately. The a, b, and c indicated that there were statistical differences among the postoperative group. Multiple comparisons were corrected by Bonferroni.

### 3.2 Relative abundance of microflora

**Figure 1:**
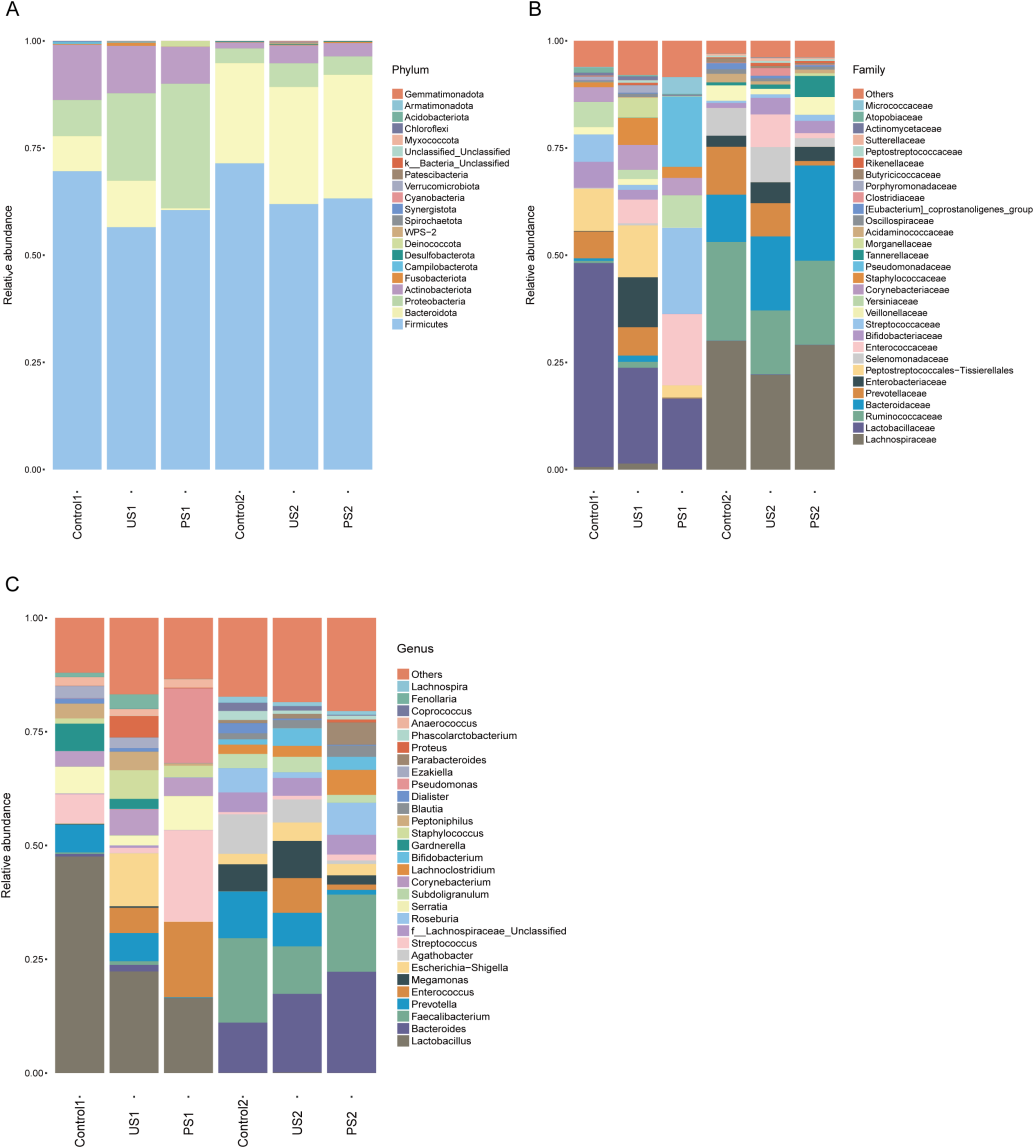
**(A–C)** Histogram of the relative abundance of the top 30 species in each group at different taxonomic levels (phylum, family, genus).

To determine the differentially representative taxa in the calculus group, the control group, and the post-calculus operation group, we analyzed the comparative abundances of the microbiota across the three groups at various taxonomic levels. At the phylum level, the average abundance of *Firmicutes* was highest in both the urethral and intestinal flora (**Figure 1A**). In addition, the contents of *Proteobacteria*, *Bacteroidota*, and *Actinobacteriota* in urine were all relatively abundant and showed differences among groups. At the family level, the abundance of *Lactobacillaceae* decreases from Control1 to US1 to PS1. The abundance of *Enterobacteriaceae* in US1 was higher than that in Control1 and PS1, and the abundance of *Enterococcaceae* and *Streptococcaceae* in PS1 was higher than that in the other two groups (**Figure 1B**). At the taxonomic level of the genus, compared with Control1, some bacterial genera in urine, such as *Lactobacillus*, *Streptococcus*, *Serratia*, and *Gardnerella*, were present at lower levels in US1, while *Enterococcus*, *Escherichia-Shigella*, *Corynebacterium*, *Staphylococcus*, and *Proteus* were more abundant in US1 (**Figure 1C**). The PS1 group showed a high abundance of *Lactobacillus*, *Enterococcus*, *Streptococcus*, and *Pseudomonas*. *Prevotella* was the most abundant in the Control2 group.

In the intestinal microbiota, apart from *Firmicutes*, *Bacteroidota* was the most abundant, far exceeding other phyla. At the family level, the relative abundance of *Lachnospiraceae*, *Ruminococcaceae*, and *Bacteroidaceae* was greater in Control2 and PS2 compared to the US2 group. At the taxonomic level of genus, the relative abundance of *Prevotella* showed a gradual decreasing trend from Control2 to US2 to PS2, while the relative abundance of *Bacteroides* showed an opposite trend. The relative abundance of *Faecalibacterium* was higher in Control2 and PS2 than in the US2 group (**Figure 1**).

### 3.3 Alpha diversity analysis

**Figure 2:**
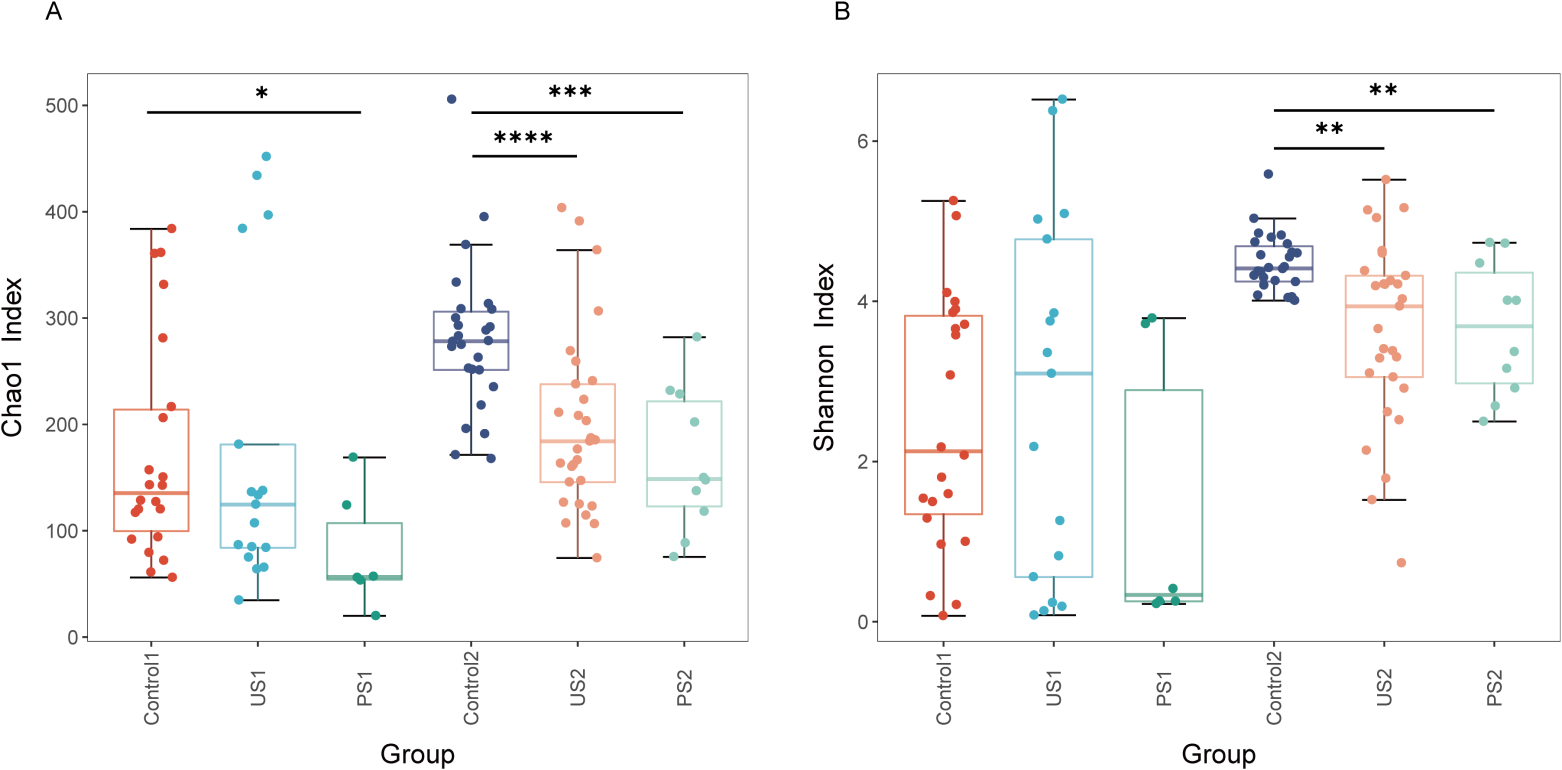
Alpha diversity across groups. Boxplots show **(A)** Chao1 Index and **(B)** Shannon Index. Groups are indicated on the x-axis; index values are on the y-axis. Inter-group differences were assessed using the Wilcoxon rank-sum test (*P<0.05; **P<0.01; ***P<0.001; ****P<0.0001).

The Goods coverage of each sample was greater than 99.5%, indicating that the coverage of each sample library was high, and the sequence of the sample represented almost all the bacterial sequences in the sample (**Supplementary Table 1**). The richness indices ACE and Chao1 indicate that the number of species in the urethral and intestinal microbiota is greater in the control group, moderate in the stone group, and least in the post-operative group. According to the analysis of Shannon and Simpson’s colony diversity index, the community diversity of Control1 was lower than that of US1 in the urine microbiota, and the community diversity of Control2 was the highest in the intestinal microbiota. In comparison to the control group and the stone group, the diversity of the urethral microbiota and intestinal microbiota in the postoperative group further declined (**Figure 2**). Compared with Control2, PS2 has a decrease in microbiome diversity and is closer to US2. After statistical analysis, there was no significant difference in urethral microbiota among the three groups (*P >*0.05), while intestinal microbiota showed significant differences (*P <*0.05) (**Table 2** and **Figure 2**).

**Table 2:** Alpha diversity analysis and statistical comparisons.

A. Urine Specimens				
Index	Control1	US1	PS1	*P*
ACE	138.24 (93.02,247.31)	117.80 (87.21,283.87)	58.59 (48.00,131.66)	0.054
Chao1	135.49 (93.47,232.61)	124.71 (79.54,282.56)	56.50 (45.07,135.24)	0.066
Shannon	2.13 (1.22,3.87)	3.10 (0.40,4.90)	0.33 (0.25,3.74)	0.410
Simpson	0.51 (0.34,0.86)	0.77 (0.08,0.93)	0.08 (0.04,0.86)	0.532

B. Fecal Specimens				
Index	Control2	US2	PS2	*P*
ACE	267.79 (239.26,305.34)	183.27 (135.32,230.84)	154.45 (111.36,206.79)	0.000
Chao1	278.19 (247.03,308.18)	184.14 (136.11,239.28)	148.68 (110.67,229.15)	0.000
Shannon	4.41 (4.23,4.72)	3.94 (2.98,4.35)	3.69 (2.86,4.54)	0.002
Simpson	0.91 (0.87,0.92)	0.86 (0.77,0.90)	0.84 (0.77,0.91)	0.012

Data are median (interquartile range, IQR). A: Alpha diversity indices in urine specimens; B: Alpha diversity indices in fecal specimens. *P*-values from Kruskal-Wallis tests (overall intergroup comparison), with statistical significance defined as *P*<0.05.

### 3.4 Beta diversity analysis

**Figure 3:**
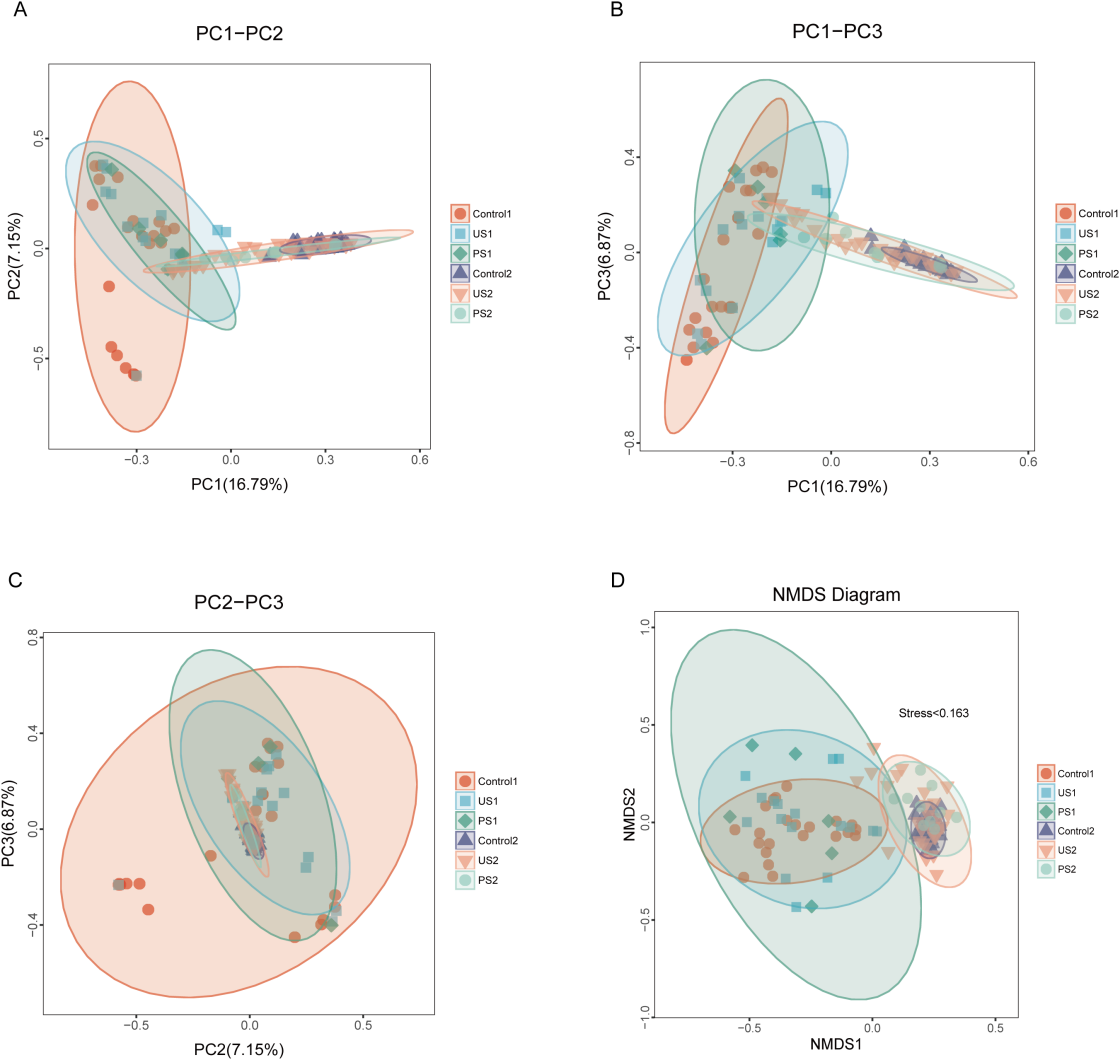
PCoA analysis and NDMS analysis. In the figure, samples belonging to the same group are represented by identical colors and shapes. **(A–C)** The percentage displayed alongside each principal coordinate indicates its contribution rate to the observed differences among samples. The distance between sample points reflects the similarity of microbial communities present in those samples. **(D)** Each point represents a sample, and the distance between the points indicates the degree of difference. Stress <0.2 indicates that NMDS can accurately reflect the degree of difference between samples.

Based on the Bray-Curtis distance matrix, principal coordinate analysis (PCoA) was conducted using the R language to assess the similarity of microbial communities within the samples. As shown in **Figure 3**, the contribution rates of PC1, PC2, and PC3 to the sample differences are 16.79%, 7.15%, and 6.87%, respectively. Urethral microbiota mainly concentrates in the upper left area of the diagram, while intestinal flora is mostly distributed in the lower right area, with significant differences in their microbial communities or metabolic characteristics. Further analysis found that the inter-group microbial differences in both urine samples and fecal samples were small. The results of non-metric multidimensional scale analysis (NMDS) showed that the stress was <0.2, indicating that NMDS could accurately reflect the degree of difference between samples. In urine and stool specimens, there were greater bacterial changes in the stone group and the postoperative group relative to the control group (**Figure 3D**).

### 3.5 Anosim inter-group difference analysis

**Figure 4:**
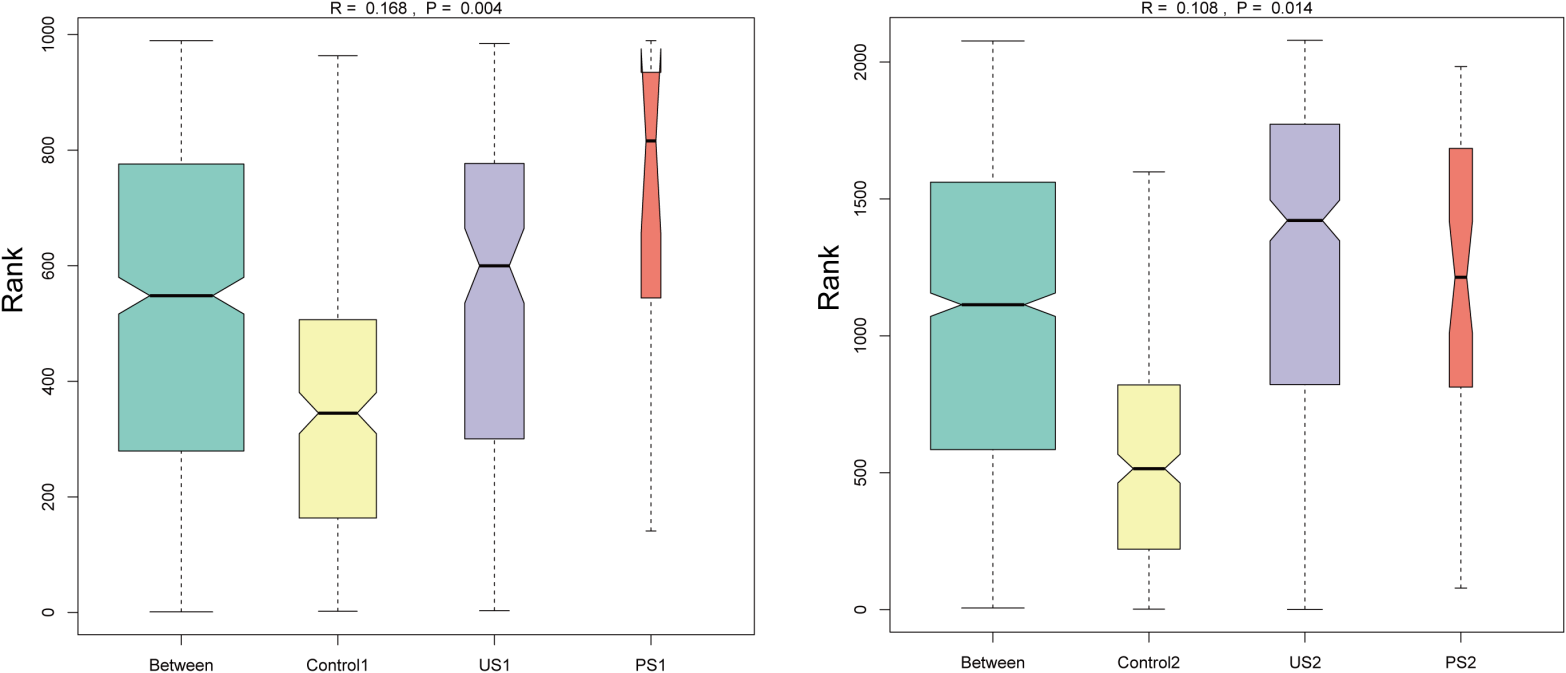
Anosim inter-group difference analysis. The vertical axis represents the rank of the distance between samples; the horizontal axis shows the results between the three groups as “Between”, and the others are the results within each group. An R value close to 1 indicates that the inter-group difference is greater than the intra-group difference, and *P <*0.05 indicates statistical significance.

In addition, Anosim was used to test whether the differences between groups were significantly greater than the differences within groups, so as to determine whether the grouping was meaningful. The ANOSIM analysis showed that the difference in intestinal microbiota between groups (R = 0.168) was small and statistically significant (*P <*0.05). The inter-group difference in urethral microbiota (R = 0.108) was also statistically significant (*P <*0.05) (**Figure 4**). This suggests that although the variances between groups are not markedly bigger than those within groups, there are considerable differences in community structure between the control, stone, and postoperative groups.

### 3.6 Differential abundance analysis

**Figure 5:**
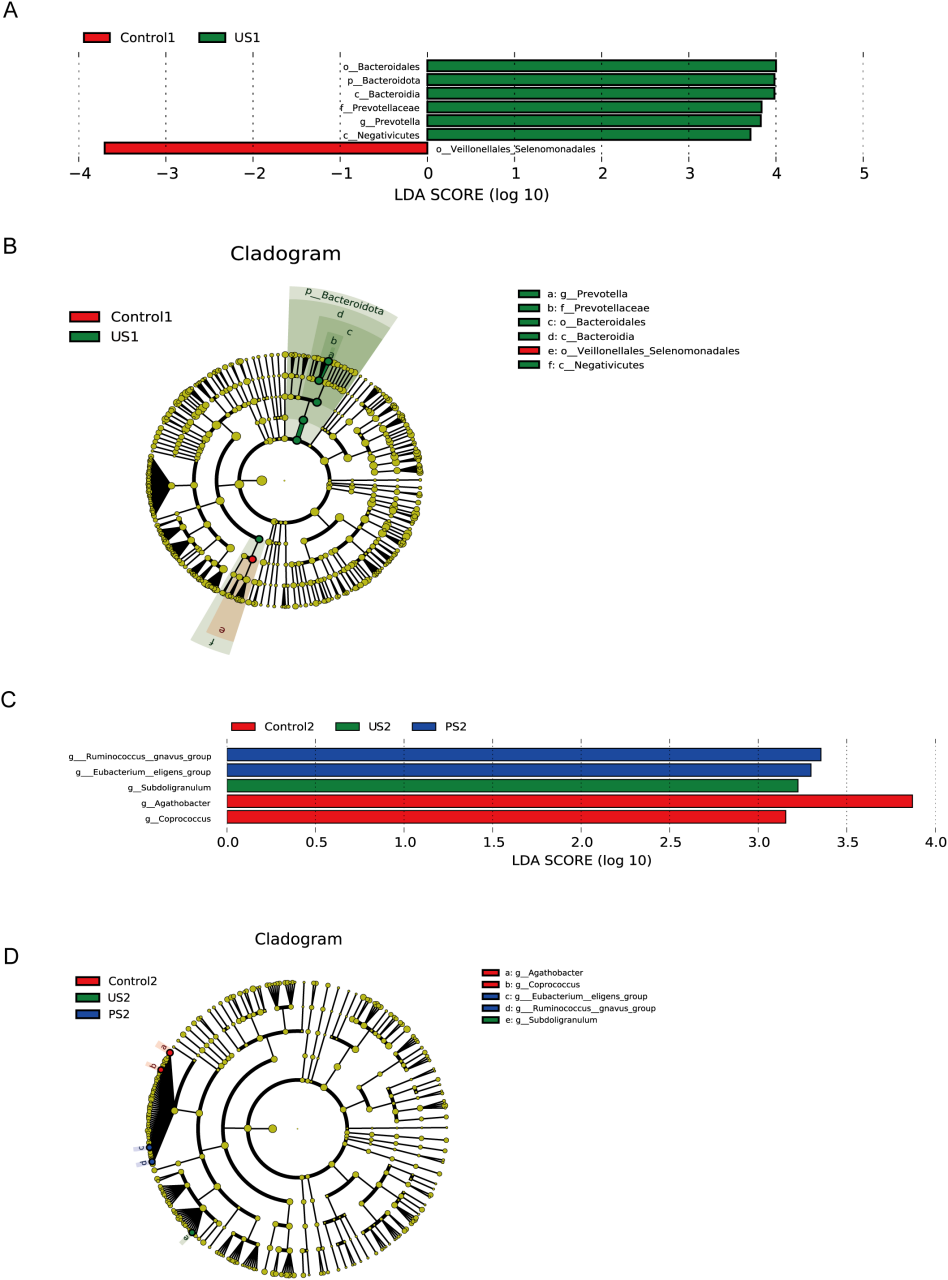
LefSe analysis. **(A, C)** The bar chart of LDA score distribution shows the significantly different species with LDA scores greater than 3. **(B, D)** Taxonomic cladogram. Different colored background areas represent groups. The concentric circles radiating outward in the figure represent the classification levels from phylum to genus. Each small circle on different classification levels represents a classification at that level, and the diameter of the small circle indicates the relative abundance.

We further used Lefse analysis to identify differentially represented taxa in different groupings of the urethra and intestine, respectively, and to obtain clade evolutionary relationships by clade plots. The results are shown in **Figure 5** (*P <*0.05, LDA >3). In the three groups of urine specimens, significant differences between the control group and the stone group were specifically reflected. The specific bacteria associated with the stone group are *Prevotella* at the genus level, *Bacteroidales* at the order level, *Bacteroidia* and *Negativicutes* at the class level, and *Bacteroidota* and *Firmicutes* at the phylum level. The specific bacteria in healthy people are *Veillonellales_Selenomonadales* within the class *Negativicutes* of the phylum *Firmicutes*. Additionally, PS1 was not included in the analysis results, which may be due to the lack of significant differences in microbial communities between this group and other groups, or these differences not passing the statistical threshold of LEfSe analysis.

In intestinal specimens, the most obvious taxa that distinguish the three taxa at the genus level are *Agathobacter* and *Coprococcus* in the control group, *Subdoligranulum* in the calculus group, and *Ruminococcus* and *Eubacterium* in the postoperative group. These bacteria belong to the class *Clostridia* of the phylum *Firmicutes*, except for *Subdoligranulum*, which is in the *Ruminococcaceae* family, and the rest are in the *Lachnospiraceae* family.

### 3.7 Wilcoxon rank-sum analysis

**Figure 6:**
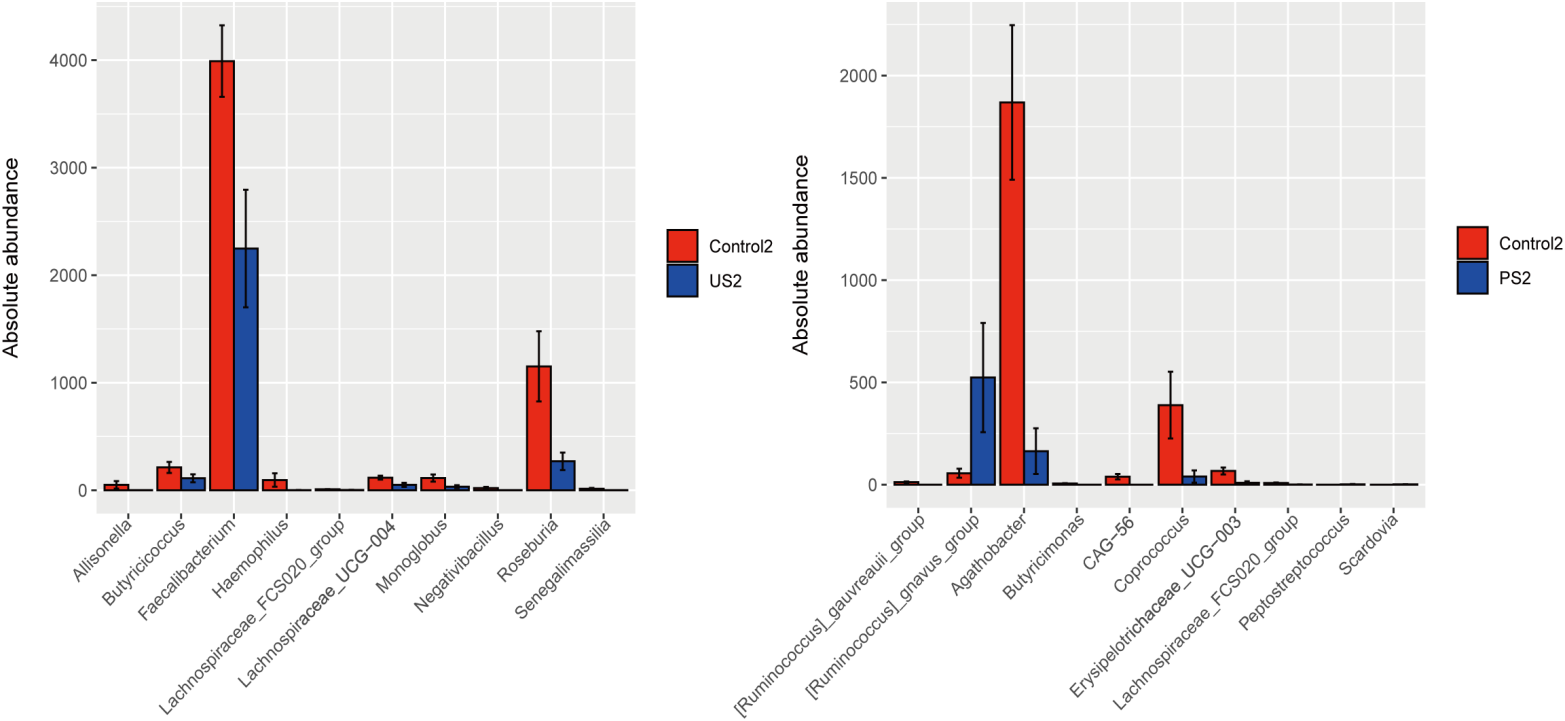
Histogram comparison between groups. The abundance distribution of the different species is shown in the figure, and the top 10 are displayed. The *P* value was corrected by using FDR.

We additionally employed the R language to perform the Wilcoxon rank sum test to assess the significant differences between the species in two groups of samples, and calculated the false discovery rate (FDR). At the genus level, the results of pairwise comparison between groups showed that Control2 was significant and credible with US2 and PS2 groups, respectively (*P <*0.05, Q <0.05), while other groups were significant but less reliable (**Supplementary Figure 2**). Through the results of histogram analysis between groups, it can be seen that the strains with significant differences between Control2 and US2 were mainly *Faecalibacterium* and *Roseburia*, etc., and the strains with significant differences between Control2 and PS2 were mainly *Ruminococcus*, *Agathobacter*, and *Coprococcus*, etc. (**Figure 6**).

## 4 Discussion

Urinary tract stones (UTS) are a common urinary system disease with a high recurrence rate. Patients with UTS may experience pain, kidney swelling, lower urinary tract symptoms, blood in urine, and psychological stress, which significantly reduces their quality of life (Raja et al., 2016; Hájková et al., 2021; Wang et al., 2024; Forbes et al., 2022). Studies have demonstrated that the urinary tract and intestinal microbiota of individuals with stones frequently differ from those of healthy individuals. The presence of pathogenic bacteria (such as *Escherichia coli*, *Enterococcus* species, and *Klebsiella pneumoniae*) in the urine of individuals with stones frequently leads to postoperative infections after stone removal surgery (Yang et al., 2022). With the increasing prevalence of stones, it is of great significance to study the role of human microbiota in stone formation and recurrence.

So far, a number of studies have demonstrated that the gut microbiota of individuals with stones is associated with short-chain fatty acids (SCFAs) (Liu et al., 2020; Chen et al., 2021). Research by Liu et al. found that SCFAs can reduce urinary oxalate and renal CaOx stones through the oxalate transporter SLC26A6 in the intestine (Liu et al., 2021a). Jin et al. showed that SCFAs prevent calcium oxalate stone formation through GPR43-dependent immunomodulatory mechanisms (Jin et al., 2021). In this study, the abundance of *Lachnospiraceae* and *Ruminococcaceae* in the intestinal microbiota of healthy people and post-stone patients was greater than that of stone patients. The members of *Lachnospiraceae* and *Ruminococcaceae* in the intestinal microbiota are primary producers of SCFAs (Biddle et al., 2013; Vacca et al., 2020). In addition, compared with the stone group, the bacteria that produced SCFAs, such as *Faecalibacterium* and *Prevotella*, were higher in the healthy controls. There were also some SCFA-producing bacteria with higher abundance in the postoperative group than in the stone group, such as *Bacteroides*, *Faecalibacterium*, *Lachnoclostridium*, and *Ruminococcus* (Akhtar et al., 2022; Hays et al., 2024). Therefore, it may provide new approaches and ideas for preventing and treating kidney stones by studying the effects of short-chain fatty acids on stones.

By comparing the relative abundance at different taxonomic levels, we identified taxa with different representativeness among the three groups. The analysis of bacterial relative abundance reveals that *Lactobacillus* richness in the urethral microbiota of the control group surpasses that of the stone and postoperative groups, while *Enterobacteriaceae* is predominant in the stone group. Studies have shown that the relative abundance of *Lactobacillus* in the urinary tract is negatively correlated with the risk of kidney stones, while the relative abundance of *Enterobacteriaceae* is positively correlated with the risk of kidney stones (Zampini et al., 2019; Chen et al., 2021; Kachroo et al., 2021). *Lactobacillus acidophilus* may inhibit the crystallization, growth, aggregation, and cell-adhesive ability of CaOx through its S-layer protein (Noonin et al., 2024). *Proteus mirabilis* is a Gram-negative bacterium belonging to *Enterobacteriaceae*, which is a common opportunistic pathogen. Recent studies have suggested that extracellular substances (such as L-lactic acid) secreted by *Lactobacillus* in the urinary tract flora can influence the pathogenicity of *Proteus mirabilis* (Szczerbiec et al., 2022, 2023). In addition, the antibacterial properties and urease inhibition ability of *Lactobacillus* can inhibit infectious urolithiasis caused by *Proteus mirabilis* (Szczerbiec et al., 2024). Apart from inducing calcium oxalate stones via flagella, PPK1, flagellin, surface elongation factor Tu, and outer membrane vesicles of *Escherichia coli* also play essential roles in the development of calcium oxalate calculi (Amimanan et al., 2017; Kanlaya et al., 2019; An et al., 2021). The abundance of lactic acid bacteria in PS1 is lower than that in US1, which is considered to be related to the shorter postoperative time, the influence of antibiotics, and the fact that the flora has not yet recovered.

*Lactobacillus* also maintains the balance of gut microbiota through various mechanisms, such as immune regulation, and a reduction in its abundance may increase the risk of stone formation (Kwak et al., 2006; Rastogi and Singh, 2022). Multi-strain probiotics (*Lactobacillus*, *Bifidobacterium*, and *O. formigenes*) have demonstrated the efficacy of degrading intestinal oxalates and regulating the microbiota in patients with urolithiasis (Taheri et al., 2024). Metabolomics evidence indicates that oxalate-degrading strains (*L. acidophilus* and *L. gasseri*) can reduce urinary oxalate excretion by using multiple carbon sources (including oxalates) (Chamberlain et al., 2019). Paradoxically, however, certain strains (*L. plantarum* and *L. brevis* strains) promote the formation of urinary calculi, possibly due to obstruction of the synthesis of antibacterial compounds in synthetic urine or a decrease in the activity of organic acids due to an increase in urine pH (Torzewska et al., 2021). In the future, the mechanism of strain-specific regulation of microbiota homeostasis in humans should be clarified to develop probiotics as adjuvant therapy for urolithiasis.

The phylum *Firmicutes* and *Bacteroidota* are the most abundant in the gut microbiota. At the genus level, the relative abundance of *Prevotella* showed a gradual decreasing trend from Control2 to US2 to PS2, while the relative abundance of *Bacteroides* showed an opposite trend. Zhao E et al (Zhao et al., 2021). found that in the gut microbiota, the levels of *Firmicutes* and *Bacteroides* were markedly increased in the kidney stone group compared to the healthy control group. Joshua M Stern et al (Stern et al., 2016). also showed a 3.4-fold higher abundance of *Bacteroides* in patients with kidney stones compared to patients without KSD (34.9 vs 10.2%; *p* = 0.001), while *Prevotella* abundance in the experimental group was only 35.4% of that in the control group (34.7 vs 12.3; *p* = 0.005), which is consistent with our analysis. A meta-analysis further confirms that *Prevotella* in the gut is strongly associated with healthy individuals, while certain types of *Prevotella* are associated with the gut microbiota of patients with urinary stone disease, a paradox that highlights the need for strain-level studies (Kachroo et al., 2021). The abundance of *Prevotella* and *Bacteroides* in the postoperative group did not fully recover to the baseline of the Control2 group, which may be related to the insufficient postoperative recovery time and the use of antibiotics. Longitudinal dynamic monitoring was considered to dynamically track the changes of flora at multiple time points after surgery. In addition, by analyzing *Prevotella* and *Bacteroides* oligotypes, De Filippis et al (De Filippis et al., 2016). revealed that dietary patterns could also specifically affect their abundance distribution and metabolic function. These studies indicate that *Prevotella* and *Bacteroide*s are associated with urinary stones, and further studies are needed to investigate their mechanisms of action.

Alpha diversity analysis showed that there were significant differences in intestinal flora, while there were no significant differences in urethral flora. The alpha diversity index of the stone group’s gut microbiota was lower than that of healthy controls, and we infer the presence of common gut microbiota characteristics that may affect stone formation. The study of Tang et al. on the intestinal microbiota of patients with kidney stones supports this inference (Tang et al., 2018). For the urethral microbiota, existing research has shown that although no substantial differences were detected in the overall microbiome between healthy individuals and stone patients, certain taxa still exhibited differential expression in the urine microbiome (Gao et al., 2022; Lee et al., 2025). Furthermore, we found that the α-diversity indices of the urethral and intestinal microbiota in the postoperative group were lower than those in the control group and the stone group. In this experiment, the postoperative group specimens were collected from clinical patients after stone surgery, and the time between specimen collection and surgery was relatively short. The decrease in postoperative α-diversity is the result of the combined effects of antibiotic exposure, mental stress, and insufficient recovery time. There are few studies on the differences in intestinal microbiota before and after kidney stone surgery. In the study by Deng et al., the α-diversity index of the intestinal microbiota before kidney stone surgery was lower than that after surgery, which may be related to the recovery of the microbiota one month after surgery (Deng et al., 2022). Importantly, we have detected a trend of reconstruction of protective microbiota (such as SCFA-producing bacteria) in the early postoperative samples, which is consistent with the microbial mechanism of kidney stone prevention (Liu et al., 2021a). Whether the results of α-diversity analysis are significant or not, it is necessary to conduct a comprehensive assessment in combination with specific research data and analysis methods to more comprehensively and accurately reveal the relationship between the urinary microbiome and stones. In the future, the dynamic recovery of the microbiota will be verified through extended postoperative follow-up.

Principal coordinates analysis (PCoA) found that the human urethral and gut microbiome may change during UUTS formation and treatment. Compared with the control group, the postoperative group was closer to the stone group in principal coordinate analysis. NDMS analysis can accurately reflect the degree of difference between sample groups, and further ANOSIM analysis confirmed that there are certain differences in community structure between different groups of stool and urine specimens. These results suggest that there are some differences in the microbial community structure of individuals with different health conditions in stool and urine specimens, and these differences are statistically significant. We found that different microbial genera were significantly associated with specific health risk factors, according to LEfSe analysis and Wilcoxon rank analysis. The abundance of the *Prevotella* genus in the urinary tract microbiota of stone patients is significantly increased and may be associated with high-risk factors for stone disease. Among the intestinal microbiota, the higher abundance of *Ruminococcus* in post-lithiasis patients and *Agathobacter* and *Coprococcus* in healthy controls may be associated with lower health risk. These microorganisms, which are significantly different in patients with stones, may be used in clinical practice to indicate the development of urinary tract stones.

This study also collected clean midstream urine for urinary chemical analysis. The K-W rank sum test showed that Ca and P were different among the three groups, and it can be considered that the calcium and phosphorus levels in the healthy group, as well as the calcium levels in the stone group, were both lower than those in the postoperative group. A study conducted in North India indicated that 24-hour urinary oxalate and calcium concentrations in individuals with nephrolithiasis were elevated compared to normal levels, while Berkemeyer’s research suggested that urine phosphorus, rather than calcium, may contribute to the pathogenesis of kidney stones (Kumar et al., 2003; Berkemeyer et al., 2007). Consequently, it can be hypothesized that the concentrations of Ca and P in the urine of urolithiasis patients undergo alterations; however, the precise patterns of these changes warrant further investigation. At present, there are few studies comparing urine chemistry among the healthy, the stone, and the postoperative group, which is the highlight of this study. The study did not conclude a significant elemental disparity between the healthy and stone groups, potentially because of the limited sample size and insufficient dietary control among individuals.

This is an experimental study aimed at determining whether there are differences in gut and urinary tract microbiota under different states of health. This study has certain advantages, involving the analysis of gut and urinary microbiota in healthy individuals, stone patients, and patients after stone surgery. It is a systematic and comprehensive study of the influence of microorganisms on the formation of kidney stones. We recruited healthy people rather than non-calculus patients as controls, excluding the effects of other disease factors on the controls. We also performed urine chemical analysis on urine specimens to analyze how the urine electrolytes of patients before and after stone surgery differ from those of healthy people. However, it must be admitted that diet, as a key regulatory factor of the intestinal microbiome, may have a confounding effect on the study. Since standardized dietary control was not implemented in the design stage of this study, we cannot rule out the influence brought by the dietary preferences of the subjects. In addition, the sample size of this study is small, especially for postoperative specimens from stone patients. This is mainly due to the difficulty in collecting clinical specimens after the discharge of patients after surgery for kidney stones. Nevertheless, our research provides preliminary evidence for future large-scale studies (n≥ 15). This limitation was unavoidable under current clinical pathways but will be addressed in future multicenter trials using remote sampling kits. Additional studies are needed to confirm these preliminary findings if further studies are to be conducted on the association between microbiota and stone diagnosis and treatment. Finally, considering the wide age range of the study participants, the microbiota and the mechanisms underlying stone formation may also vary among patients of different age groups. Thus, in subsequent research, we will conduct an in-depth analysis of microbiota differences across various age and gender subgroups of patients with calculi.

In this study, the abundance of *Lactobacillus* in the urinary tract was higher in healthy people, while *Enterobacteriaceae* was higher in the stone group. *Bacteroides* and *Prevotella* in the intestine showed variations among distinct groups. LEfSe analysis and Wilcoxon rank sum analysis found that multiple microbial genera were significantly associated with stone risk factors. Notably, we observed that some intestinal microbiota involved in the production of short-chain fatty acids (SCFAs) exhibit distinct distributions among different groups. This finding provides innovative insights and methodologies for the prevention and management of stone disease. We also performed urine chemistry analysis on the urine of the participants, speculating that Ca and P may play a crucial role in the formation of kidney stones. In conclusion, we suggest that changes in gut and urinary microbiota composition are associated with stone status and that these microbial signatures may serve as potential biomarkers of stone risk. In clinical intervention, regulating the stability of microbiota may become an auxiliary means, and it is necessary to avoid the increased risk of drug resistance and recurrence caused by the abuse of antibiotics.

## 5 Conclusion

The urinary tract microbiota and gut microbiota have different compositions and diversity in patients with UUTS, healthy individuals, and post-stone surgery populations. *Enterobacteriaceae* and *Bacteroides* are more abundant in patients with stones. The increased abundance of *Lactobacillus*, *Lachnospiraceae*, *Rumenococcaceae*, *Faecalibacterium*, and *Prevotella* is associated with a reduced risk of kidney stones. LEfSe and Wilcoxon rank sum analysis showed that *Prevotella* in the urethra and *Ruminococcus*, *Agathobacter*, and *Coprococcus* in intestinal flora could be used to classify healthy people, stone patients, and the post-stone population. In the future, it is considered to improve the microbiota of UUTS patients through diet or probiotics to prevent and treat stones, and enhance the quality of life for patients.

## Data Availability

The datasets presented in this study can be found in online repositories. The names of the repository/repositories and accession numbers can be found below: https://www.ncbi.nlm.nih.gov/, PRJNA1259181.
