# Supplementary material for: Dysbiosis of gut and urinary microbiota in urolithiasis patients and post-surgical cases
Source: Front Cell Infect Microbiol. 2025 Aug 13;15:1633783. doi: 10.3389/fcimb.2025.1633783 (PMC12380916; doi:10.3389/fcimb.2025.1633783)
Supplement: Supplementary file 1 [file Table1.docx]

Supplementary Material

# Supplementary Tables

# Supplementary Table 1. Alpha diversity analysis

| group | sample ID | ace | chao1 | shannon | simpson | goods_coverage |
| --- | --- | --- | --- | --- | --- | --- |
| control1 | 1 | 89.961 | 94 | 3.077 | 0.742 | 0.999 |
| control1 | 2 | 152.586 | 150.353 | 3.897 | 0.88 | 0.999 |
| control1 | 3 | 150.425 | 143 | 1.801 | 0.486 | 0.998 |
| control1 | 4 | 116.039 | 142.5 | 3.994 | 0.883 | 0.999 |
| control1 | 5 | 109.293 | 120.25 | 3.857 | 0.871 | 0.999 |
| control1 | 6 | 129.136 | 117 | 1.291 | 0.303 | 0.999 |
| control1 | 7 | 94.039 | 91.875 | 1.539 | 0.491 | 0.999 |
| control1 | 8 | 88.332 | 79.429 | 0.965 | 0.295 | 0.999 |
| control1 | 9 | 62.309 | 61 | 0.074 | 0.012 | 0.999 |
| control1 | 10 | 372.37 | 383.848 | 3.709 | 0.71 | 0.997 |
| control1 | 11 | 322.741 | 331.345 | 5.065 | 0.94 | 0.997 |
| control1 | 12 | 279.695 | 281.077 | 3.655 | 0.729 | 0.999 |
| control1 | 13 | 204.404 | 206.048 | 3.577 | 0.81 | 0.999 |
| control1 | 14 | 236.51 | 216.459 | 2.077 | 0.525 | 0.997 |
| control1 | 15 | 368.996 | 360.522 | 4.106 | 0.854 | 0.997 |
| control1 | 16 | 342.269 | 361.435 | 5.254 | 0.934 | 0.998 |
| control1 | 17 | 65.612 | 56 | 1 | 0.473 | 0.999 |
| control1 | 18 | 133.989 | 128.476 | 1.497 | 0.354 | 0.999 |
| control1 | 19 | 88.322 | 72 | 0.212 | 0.039 | 0.999 |
| control1 | 20 | 142.492 | 127.176 | 1.595 | 0.439 | 0.999 |
| control1 | 21 | 123.781 | 120.067 | 0.322 | 0.054 | 0.999 |
| control1 | 22 | 163.573 | 157.059 | 2.178 | 0.465 | 0.998 |
| US1 | 1 | 85.533 | 84.8 | 3.357 | 0.846 | 0.999 |
| US1 | 2 | 91.35 | 86.667 | 0.559 | 0.113 | 0.999 |
| US1 | 3 | 113.442 | 107.154 | 3.751 | 0.88 | 0.999 |
| US1 | 4 | 420.192 | 433.824 | 6.377 | 0.97 | 0.997 |
| US1 | 5 | 426.271 | 451.667 | 6.52 | 0.974 | 0.997 |
| US1 | 6 | 387.859 | 384 | 5.023 | 0.921 | 0.997 |
| US1 | 7 | 93.233 | 84.077 | 0.19 | 0.031 | 0.999 |
| US1 | 8 | 368.674 | 396.621 | 5.092 | 0.945 | 0.997 |
| US1 | 9 | 117.797 | 137.667 | 0.82 | 0.187 | 0.999 |
| US1 | 10 | 135.649 | 136.375 | 4.775 | 0.94 | 0.999 |
| US1 | 11 | 132.56 | 124.714 | 3.098 | 0.772 | 0.999 |
| US1 | 12 | 58.608 | 65.5 | 1.26 | 0.361 | 0.999 |
| US1 | 13 | 40.349 | 34.625 | 0.081 | 0.013 | 1 |
| US1 | 14 | 199.059 | 181.118 | 2.184 | 0.522 | 0.998 |
| US1 | 15 | 139.728 | 133.5 | 3.852 | 0.86 | 0.999 |
| US1 | 16 | 76.278 | 63.929 | 0.237 | 0.044 | 0.999 |
| US1 | 17 | 88.891 | 75 | 0.134 | 0.023 | 0.999 |
| PS1 | 1 | 117.476 | 124 | 3.718 | 0.857 | 0.999 |
| PS1 | 2 | 59.149 | 56 | 0.224 | 0.044 | 0.999 |
| PS1 | 3 | 57.541 | 57 | 0.412 | 0.113 | 0.999 |
| PS1 | 4 | 174.201 | 168.957 | 3.789 | 0.886 | 0.998 |
| PS1 | 5 | 58.033 | 53.429 | 0.256 | 0.045 | 0.999 |
| PS1 | 6 | 19.36 | 20 | 0.254 | 0.056 | 1 |
| control2 | 1 | 347.216 | 395.053 | 4.715 | 0.919 | 0.997 |
| control2 | 2 | 267.894 | 278.5 | 4.053 | 0.857 | 0.997 |
| control2 | 3 | 265.741 | 274.955 | 4.2 | 0.873 | 0.998 |
| control2 | 4 | 195.628 | 191.05 | 4.044 | 0.867 | 0.998 |
| control2 | 5 | 267.901 | 262.778 | 4.823 | 0.923 | 0.998 |
| control2 | 6 | 317.281 | 308.5 | 4.295 | 0.88 | 0.997 |
| control2 | 7 | 282.071 | 273 | 4.321 | 0.865 | 0.998 |
| control2 | 8 | 202.16 | 195.929 | 4.418 | 0.908 | 0.998 |
| control2 | 9 | 220.461 | 218 | 4.429 | 0.916 | 0.998 |
| control2 | 10 | 288.932 | 292.87 | 4.577 | 0.917 | 0.997 |
| control2 | 11 | 303.432 | 300 | 4.376 | 0.871 | 0.997 |
| control2 | 12 | 185.147 | 171.316 | 4.074 | 0.864 | 0.998 |
| control2 | 13 | 242.769 | 252.688 | 4.797 | 0.928 | 0.998 |
| control2 | 14 | 279.556 | 277.875 | 4.738 | 0.929 | 0.997 |
| control2 | 15 | 266.624 | 283 | 4.365 | 0.896 | 0.997 |
| control2 | 16 | 285.89 | 288.44 | 4.55 | 0.922 | 0.997 |
| control2 | 17 | 172.972 | 167.667 | 4.009 | 0.895 | 0.998 |
| control2 | 18 | 368.644 | 368.903 | 5.585 | 0.945 | 0.997 |
| control2 | 19 | 311.059 | 313.357 | 4.609 | 0.907 | 0.997 |
| control2 | 20 | 267.686 | 308.077 | 4.243 | 0.875 | 0.997 |
| control2 | 21 | 242.167 | 291.5 | 4.019 | 0.859 | 0.998 |
| control2 | 22 | 528.053 | 505.517 | 5.033 | 0.912 | 0.995 |
| control2 | 23 | 240.938 | 235.125 | 4.6 | 0.913 | 0.998 |
| control2 | 24 | 234.238 | 251.588 | 4.256 | 0.875 | 0.998 |
| control2 | 25 | 262.429 | 251 | 4.405 | 0.915 | 0.997 |
| control2 | 26 | 323.281 | 333.517 | 4.848 | 0.924 | 0.997 |
| US2 | 1 | 270.785 | 306.333 | 4.319 | 0.887 | 0.997 |
| US2 | 2 | 237.27 | 240.8 | 3.938 | 0.872 | 0.998 |
| US2 | 3 | 171.741 | 163.316 | 2.138 | 0.491 | 0.998 |
| US2 | 4 | 195.141 | 203.235 | 3.053 | 0.776 | 0.998 |
| US2 | 5 | 269.959 | 259.143 | 4.599 | 0.901 | 0.998 |
| US2 | 6 | 192.784 | 187.188 | 4.213 | 0.905 | 0.998 |
| US2 | 7 | 186.901 | 184.136 | 3.303 | 0.82 | 0.998 |
| US2 | 8 | 110.061 | 123 | 2.518 | 0.666 | 0.999 |
| US2 | 9 | 135.313 | 125 | 1.789 | 0.481 | 0.998 |
| US2 | 10 | 164.855 | 176.6 | 4.214 | 0.884 | 0.999 |
| US2 | 11 | 135.32 | 145.625 | 4.191 | 0.91 | 0.999 |
| US2 | 12 | 352.031 | 363.897 | 5.165 | 0.946 | 0.996 |
| US2 | 13 | 142.38 | 147 | 2.618 | 0.722 | 0.998 |
| US2 | 14 | 213.603 | 237.769 | 4.63 | 0.923 | 0.998 |
| US2 | 15 | 161.764 | 166.462 | 3.405 | 0.831 | 0.998 |
| US2 | 16 | 224.408 | 223.286 | 4.256 | 0.897 | 0.998 |
| US2 | 17 | 269.395 | 269 | 5.041 | 0.933 | 0.998 |
| US2 | 18 | 207.813 | 211.077 | 4.38 | 0.901 | 0.998 |
| US2 | 19 | 131.13 | 126.6 | 3.102 | 0.77 | 0.999 |
| US2 | 20 | 183.265 | 185.188 | 4.027 | 0.85 | 0.998 |
| US2 | 21 | 85.877 | 74.375 | 2.913 | 0.824 | 0.999 |
| US2 | 22 | 211.34 | 208.15 | 4.209 | 0.866 | 0.998 |
| US2 | 23 | 399.062 | 391 | 5.517 | 0.955 | 0.996 |
| US2 | 24 | 129.493 | 106.429 | 0.733 | 0.185 | 0.999 |
| US2 | 25 | 117.637 | 107.077 | 1.521 | 0.478 | 0.999 |
| US2 | 26 | 159.206 | 162 | 3.289 | 0.791 | 0.998 |
| US2 | 27 | 383.658 | 403.516 | 5.137 | 0.928 | 0.996 |
| US2 | 28 | 154.486 | 160.2 | 3.656 | 0.857 | 0.998 |
| US2 | 29 | 124.057 | 114.526 | 3.38 | 0.837 | 0.999 |
| PS2 | 1 | 127.215 | 137.429 | 3.16 | 0.781 | 0.999 |
| PS2 | 2 | 200.445 | 202 | 4.722 | 0.905 | 0.998 |
| PS2 | 3 | 196.562 | 231.667 | 4.474 | 0.926 | 0.998 |
| PS2 | 4 | 91.483 | 88.462 | 2.692 | 0.75 | 0.999 |
| PS2 | 5 | 76.055 | 75.429 | 2.499 | 0.64 | 0.999 |
| PS2 | 6 | 225.815 | 228.316 | 4.731 | 0.929 | 0.998 |
| PS2 | 7 | 165.099 | 147.556 | 4.009 | 0.885 | 0.999 |
| PS2 | 8 | 143.801 | 149.8 | 3.369 | 0.842 | 0.998 |
| PS2 | 9 | 294.21 | 282.033 | 4.01 | 0.84 | 0.997 |
| PS2 | 10 | 117.988 | 118.077 | 2.916 | 0.803 | 0.999 |

**Supplementary Table 2. Effect Sizes and Mean Differences of Alpha-Diversity Metrics Between Groups**

| Metric | Comparison Group | Cohen's d | Hedges' g(95% CI) | Raw Mean Difference | Bootstrap Mean Difference (R=5000) | Bootstrap 95% BCa CI | |
| --- | --- | --- | --- | --- | --- | --- | --- |
|  |  |  |  |  |  | lower | upper |
| Chao1 | Control1 vs PS1 | 0.96 | 0.932(0.011,1.835) | 92.87138 | 92.80864 | 22.95202 | 138.7069 |
|  | US1 vs PS1 | 0.749 | 0.721(-0.209,1.636) | 95.46927 | 95.80155 | 76.24906 | 238.6012 |
|  | Control2 vs PS2 | 1.63 | 1.594(0.778,2.391) | 114.3156 | 113.7947 | 78.18285 | 173.8224 |
|  | US2 vs PS2 | 0.41 | 0.402(-0.311,1.109) | 32.9551 | 32.65178 | 3.675184 | 101.4602 |
| Shannon | Control1 vs PS1 | 0.644 | 0.625(-0.273,1.512) | 1.046106 | 1.05618 | 1.105749 | 3.118939 |
|  | US1 vs PS1 | 0.618 | 0.596(-0.326,1.504) | 1.340775 | 1.364627 | 0.6940388 | 3.838906 |
|  | Control2 vs PS2 | 1.535 | 1.501(0.695,2.289) | 0.8180308 | 0.8188099 | 0.1385254 | 1.111012 |
|  | US2 vs PS2 | -0.026 | -0.026(-0.73,0.678) | -0.02875172 | -0.02866932 | -0.3733985 | 0.9541121 |

**Supplementary Table 3. Adonis analysis of beta-diversity in male urinary and fecal microbiota across age subgroups**

| **Sample** | **Source** | **Df** | **SumOfSqs** | **R2** | **F** | ***P*** |
| --- | --- | --- | --- | --- | --- | --- |
| Urine | Model | 2 | 0.869 | 0.190 | 1.055 | 0.358 |
|  | Residual | 9 | 3.704 | 0.810 | - | - |
|  | Total | 11 | 4.573 | - | - | - |
| Feces | Model | 2 | 0.671 | 0.066 | 0.850 | 0.734 |
|  | Residual | 24 | 9.471 | 0.934 | - | - |
|  | Total | 26 | 10.142 | - | - | - |

Young [≤40 yr], Middle-aged [41-60 yr], Elderly [>60 yr]).

**Supplementary Table 4. ANOSIM assessment of microbial community dissimilarity among male age cohorts**

| **Sample** | **R-value** | ***P***-value |
| --- | --- | --- |
| Urine | 0.017 | 0.402 |
| Feces | -0.017 | 0.535 |

# Supplementary Figures


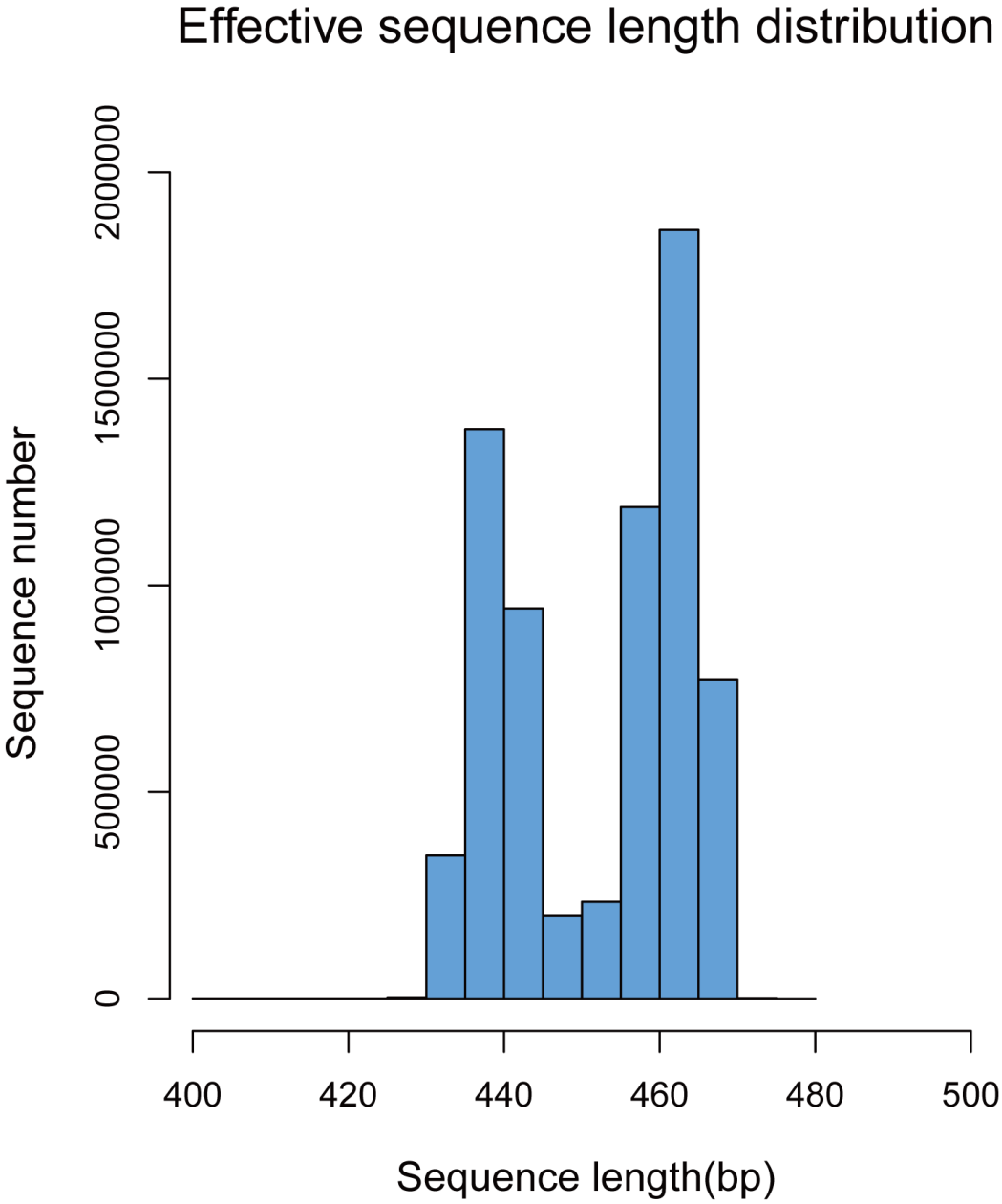


**Supplementary Figure 1.** Effective sequence length distribution statistics chart. The abscissa is the sequence length (bp), and the ordinate is the number of sequences of different lengths.


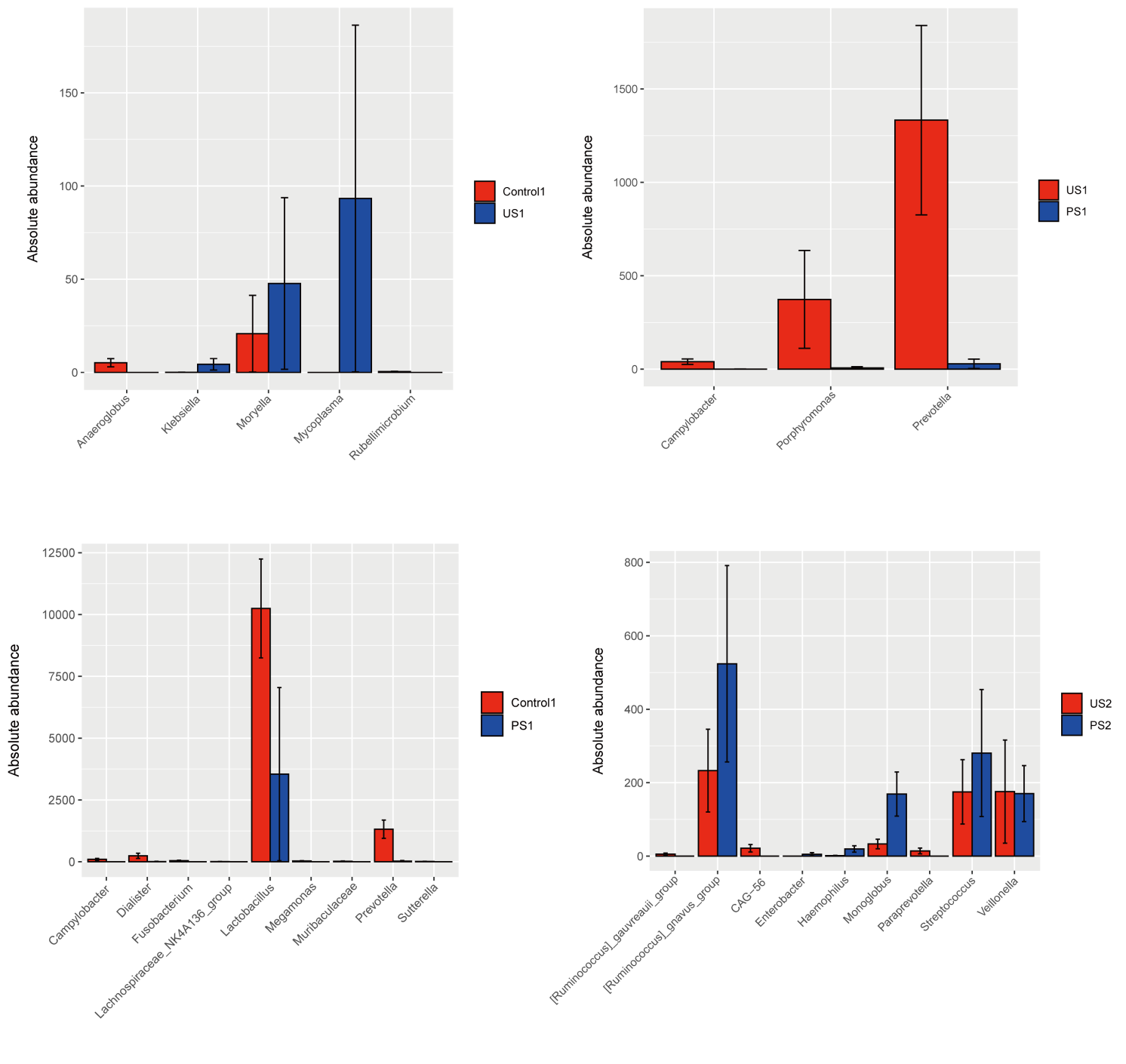


**Supplementary Figure 2.** Histogram comparison between groups. The abundance distribution of the different species is shown in the figure, and the top 10 is displayed.


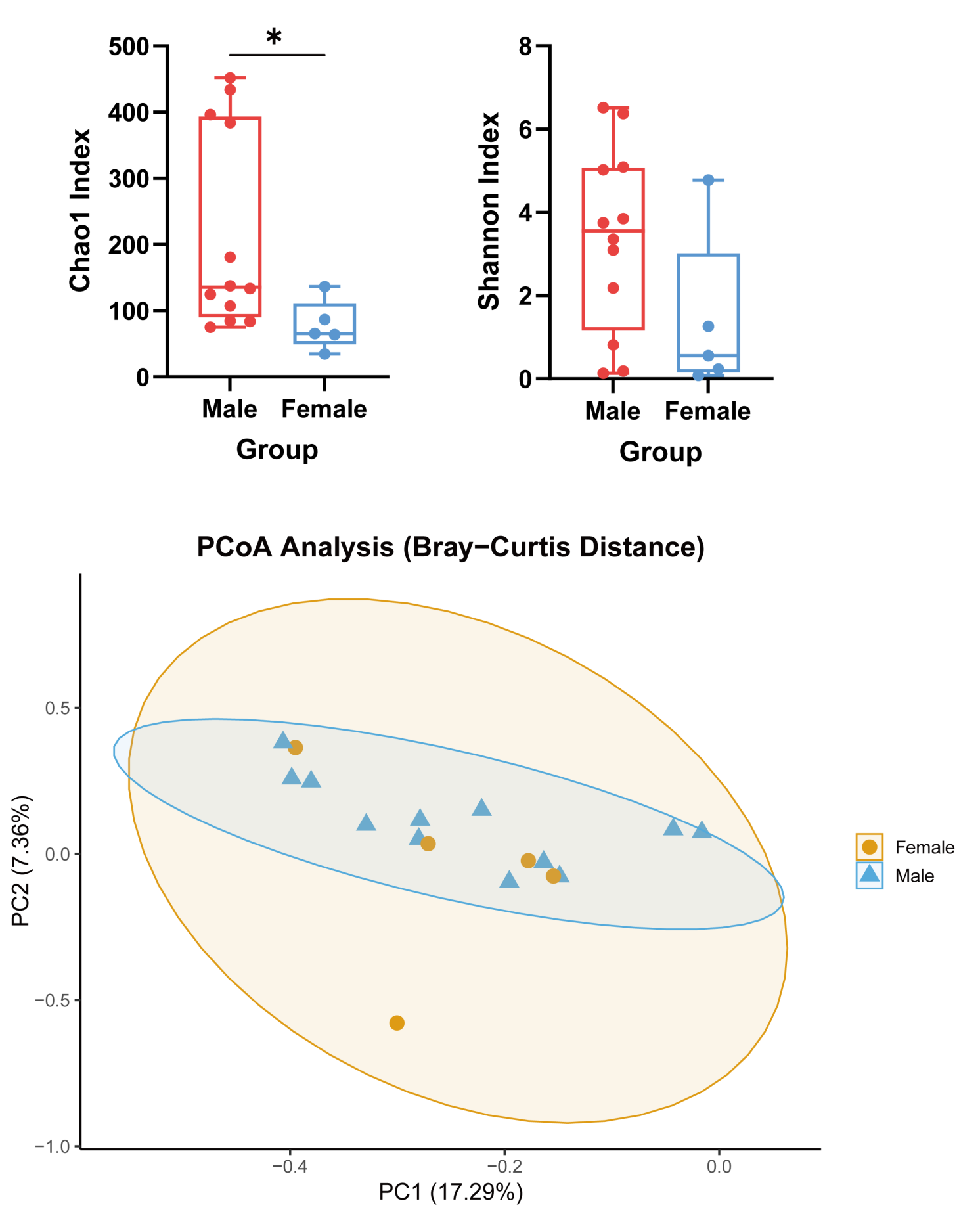


**Supplementary Figure 3.** Comparison of alpha diversity and PCoA analysis of urethra microbiota between male and female. **P*<0.05, Wilcoxon rank-sum test.
