# Supplementary material for: Dysbiosis of gut and urinary microbiota in urolithiasis patients and post-surgical cases
Source: Front Cell Infect Microbiol. 2025 Aug 13;15:1633783. doi: 10.3389/fcimb.2025.1633783 (PMC12380916; doi:10.3389/fcimb.2025.1633783)
Supplement: Supplementary file 2 [file Table2.docx]

# Table 1. Statistical analysis of urine chemical elements in healthy, stone and postoperative groups.

|  | Control (N=31) | US (N=38) | PS (N=12) | H | *P* |
| --- | --- | --- | --- | --- | --- |
| K | 36.20(8.49,60.04) | 24.09(12.68,28.95) | 19.15(10.02,39.89) | 2.388 | 0.303 |
| Na | 139.00(38.00,196.00) | 107.00(67.50,137.75) | 90.00(78.75,133.75) | 1.793 | 0.408 |
| Cl | 109.50(33.00,183.40) | 81.25(57.30,125.08) | 83.30(50.73,107.35) | 2.249 | 0.325 |
| Ca | 2.36(0.92,3034)^a^ | 1.71(1.12,3.88)^b^ | 4.33(2.74,5.49) | 8.141 | 0.017 |
| P | 13.37(4.26,14.73)^c^ | 13.35(9.88,15.29) | 18.69(13.50,25.48) | 10.214 | 0.006 |
| Mg | 1.77(0.75,3.03) | 2.27(1.25,2.91) | 2.61(2.07,3.30) | 3.363 | 0.186 |

The median and quartile of each group, the test statistic H, and P values of the Kruskal-Wallis test were calculated separately. a, b, and c indicated that there were statistical differences with the postoperative group. All comparisons were corrected by Bonferroni.
