# Supplementary material for: Dysbiosis of gut and urinary microbiota in urolithiasis patients and post-surgical cases
Source: Front Cell Infect Microbiol. 2025 Aug 13;15:1633783. doi: 10.3389/fcimb.2025.1633783 (PMC12380916; doi:10.3389/fcimb.2025.1633783)
Supplement: Supplementary file 3 [file Table3.docx]

# Table 2. Alpha diversity analysis and statistical comparisons

A

|  | Control1 | US1 | PS1 | *P* |
| --- | --- | --- | --- | --- |
| ACE | 138.24(93.02,247.31) | 117.80(87.21,283.87) | 58.59(48.00,131.66) | 0.054 |
| Chao1 | 135.49(93.47,232.61) | 124.71(79.54,282.56) | 56.50(45.07,135.24) | 0.066 |
| Shannon | 2.13(1.22,3.87) | 3.10(0.40,4.90) | 0.33(0.25,3.74) | 0.410 |
| Simpson | 0.51(0.34,0.86) | 0.77(0.08,0.93) | 0.08(0.04,0.86) | 0.532 |
| B |  |  |  |  |
|  | Control2 | US2 | PS2 | *P* |
| ACE | 267.79(239.26,305.34) | 183.27(135.32,230.84) | 154.45(111.36,206.79) | 0.000 |
| Chao1 | 278.19(247.03,308.18) | 184.14(136.11,239.28) | 148.68(110.67,229.15) | 0.000 |
| Shannon | 4.41(4.23,4.72) | 3.94(2.98,4.35) | 3.69(2.86,4.54) | 0.002 |
| Simpson | 0.91(0.87,0.92) | 0.86(0.77,0.90) | 0.84(0.77,0.91) | 0.012 |

The first three columns present the medians and interquartile ranges for the three groups across the four indicators, with P-values calculated using the Kruskal-Wallis rank-sum test.
